# Enhancing Telemedicine Communication for Improved Outpatient Pediatric Trauma Care

**DOI:** 10.3390/children11091120

**Published:** 2024-09-12

**Authors:** Nariman Mokhaberi, Benjamin Schoof, André Strahl, Konrad Reinshagen, Kristofer Wintges

**Affiliations:** 1 Department of Pediatric Surgery, University Medical Center Hamburg-Eppendorf, 20251 Hamburg, Germany; 2 Department of Trauma Surgery and Orthopedics, University Medical Center Hamburg-Eppendorf, 20251 Hamburg, Germany; 3 Department of Trauma Surgery and Orthopedics, Division of Orthopedics, University Medical Center Hamburg-Eppendorf, 20251 Hamburg, Germany

**Keywords:** telemedicine, pediatric trauma, survey, telehealth, children

## Abstract

Introduction. Pediatric traumatology is a complex field that requires a comprehensive understanding of physeal development, remodeling potential, and the ossification process in order to ensure appropriate patient treatment. The objective of this study was to assess the willingness of practicing physicians to participate in a telemedicine collaboration aimed at enhancing the exchange between the outpatient and inpatient sectors and promoting the digitalization of the pediatric sector. This is in response to the growing significance of digitalization in the medical field. Methods. A survey consisting of 15 items was sent to 800 practicing trauma surgeons, pediatric surgeons, and pediatricians within a 100 km radius of Hamburg, Germany. The survey included questions about the respondents’ professional experience and telemedicine experience, as well as inquiries about possible telemedicine collaborations. Results. The response rate was 19.3%. Less than half of the participants already used telemedicine in daily practice. In general, 75% of respondents expressed an interest in collaborating with the inpatient sector. The most common reasons for hospital referral were the need for surgery, inadequate treatment of children in practice and co-assessment. The majority were in favor of flexible communication, either via video telephony, imaging applications like or messaging applications. Conclusions. The study revealed a high level of interest in telemedicine collaboration. Information exchange should be tailored to individual needs, with practitioners requiring a versatile and personalized approach that includes imaging. Strict enforcement of data protection regulations is essential. Further research is needed to evaluate the effectiveness of telemedicine collaboration in the treatment of pediatric trauma in both hospital and outpatient settings.

## 1. Introduction

In pediatric traumatology, most injuries that occur during the years of growth can be treated conservatively [1,2]. A detailed understanding of the development and closure of the physis, the remodeling potential of the bone and the ossification process is necessary to choose the appropriate treatment [3]. Physiological findings in pediatric trauma can often be misinterpreted as fractures or pathology. For physicians with limited experience in pediatric traumatology the interpretation of X-rays can be challenging. If the treatment is not tailored to the patient’s skeletal age and individual need, it may result in physeal damage, growth retardation and impaired motor function [4,5].

Consequently, based on our experience, requests for joint assessment are frequently made in many new cases presented to the emergency department and outpatient clinics. With rising patient numbers and diminishing staff resources, this results in longer waiting times and an increased higher workload for the staff [6]. In addition, co-assessment may result in repeating examinations, such as X-rays, or unnecessary dressing changes, both of which can be associated with anxiety and pain for the patient [7].

Digitalization is now having an increasing impact on the medical field. Telemedicine is being used in a variety of ways, including telementoring during surgery, providing remote care and use in follow-up visits [8,9,10]. Nevertheless, telemedicine has not been widely adopted in pediatrics due to the inexperience of physicians, organizational challenges, and the limitations posed by the absence of physical examination [11]. However, today’s generation of parents, who are experienced in using digital assets and tools, are well-suited to be users of telemedicine. These parents belong to a generation often referred to as ‘tech natives’ or ‘digital natives’. For them, technology is ubiquitous, intuitive and familiar [12].

The contact restrictions resulting from the SARS-CoV-2 pandemic caused significant changes in hospital routines, leading to significant increase in the use of telemedicine in patient care worldwide. From January 2020 to August 2021, a total of 53 studies in adult medicine were included in a review of patient satisfaction with the use of telemedicine during the SARS-CoV-2 pandemic. A generally high level of acceptance was observed. Three studies also indicated a lower no-show rate. The use of telemedicine has also been found to enhance the effectiveness and efficiency of the health care system [13]. A previous study on pediatric patients, regarding patients with congenital malformations or malignant tumors, demonstrated that telemedicine follow-up was almost universally accepted by patients, parents and physicians. The follow-up care for these complex diseases also resulted in a high level of satisfaction for both patients and physicians, along with savings in terms of time and finances [10].

Since nearly all hospitals in Germany utilize digital radiology. The professional exchange of imaging data via “The Picture Archiving and Communication System” (PACS) is already well-established within the hospital sector in Germany [14]. It is often used for consultation regarding traumatological patients [15]. Furthermore, current data indicate a broad acceptance of medical apps in everyday clinical practice among adult trauma patients [16].

Although there have been numerous promising approaches to implement telemedicine in pediatric trauma care, these processes have not yet been regularly established in outpatient pediatric traumatology due to barriers faced by both physicians and patients. Patients reported several concerns, including misconceptions and fear, as well as technical incompetence and the potential need to use new devices. Physicians, for their part, cited a number of issues, including licensure issues, reimbursement for telemedicine services, and malpractice liability [17,18].

The objective of this study was to ascertain the willingness of general practitioners to participate in telemedical collaboration within the field of pediatric traumatology and to identify potential factors that may impede this willingness. Improving such cooperation could significantly enhance the collaboration between outpatient and inpatient sectors and promote digitalization in pediatric traumatology.

## 2. Methods

A 15-item survey was designed by the Department of Pediatric Surgery and Traumatology of the University Medical Center Hamburg-Eppendorf. The questionnaire was developed independently with regard to everyday clinical practice in our hospital (Supplemental Material S1). Its 15 questions were designed to elicit as many responses as possible, as it has been reported that quality, reliability and response rate can be severely affected with an increasing number of questions. This can result in a tendency for participants to speed through the questions [19].

**Figure 1:**
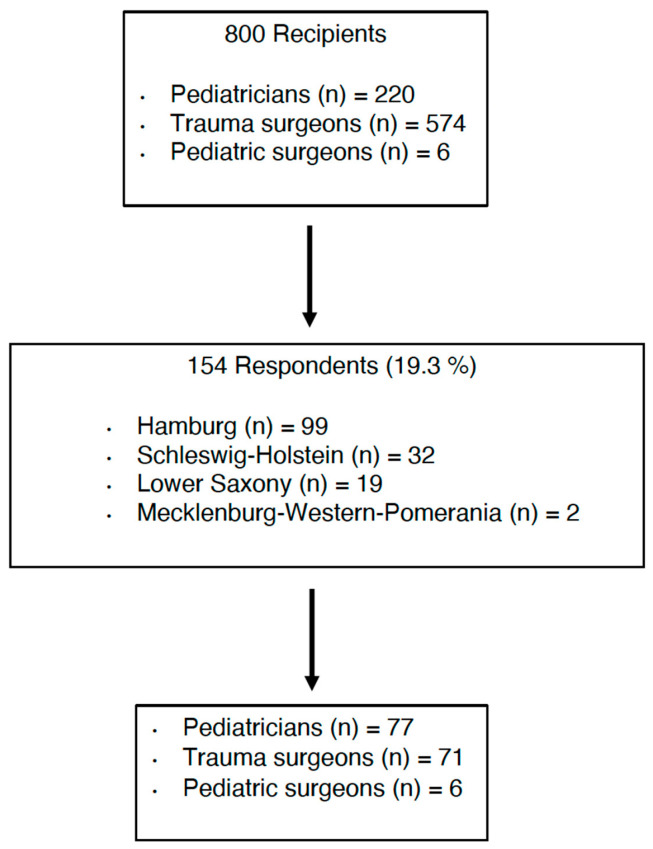
Flowchart of participant distribution.

This questionnaire, featuring both single-choice and multiple-choice questions, gathered information about the recipient’s place of residency and professional experience, particularly in pediatric traumatology. It also addressed their previous experience with telemedicine and their willingness to participate in telemedicine collaboration. The anonymous mail survey was sent in January 2023 to 800 practicing trauma surgeons, pediatric surgeons and pediatricians within a 100 km radius of Hamburg, Germany. All practitioners were registered in our department’s database (Figure 1).

The statistical program SSPS 29.0 (SPSS, Chicago, IL, USA) was used for statistical analyses. The significance level was set at *p* < 0.05 for all statistical analyses. Descriptive statistics are presented in both total numbers and relative percentages. Differences between groups were calculated using the Chi2-test. Missing data were excluded from the statistical calculation.

## 3. Results

### 3.1. Demographic and Work Specific Characteristics

Out of the 800 physicians contacted, 19.3% (154/800) responded to the survey. In total, 61.7% were male and 36.4% were female. The majority were between 40 and 50 years old (51.9%), followed by 27.3% of participants being over 60 years old.

The majority of physicians (50.0%) were pediatricians, with trauma surgeons comprising the next largest group. The majority of respondents (63.6%) had more than 20 years of professional experience, while 33.1% had worked for 10 to 20 years and 3.2% had worked for 5 to 10 years.

The collective was divided almost equally between private and group practices (42.9% vs. 41.6%). In total, 14.9% of practitioners were employed in outpatient health centers.

Key demographic and work-related specifics are detailed in Table 1.

**Figure 2:**
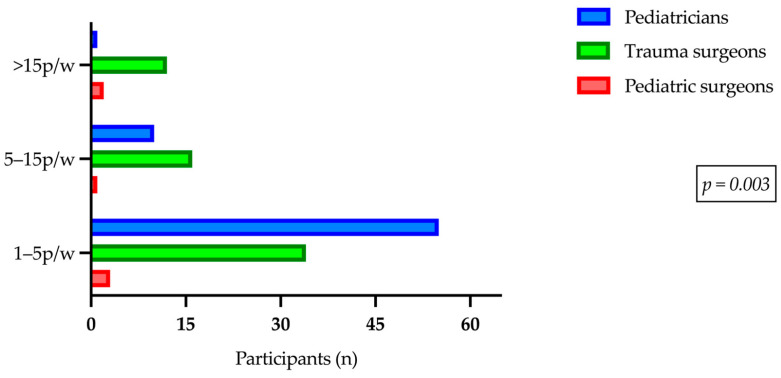
Number of pediatric trauma patients treated per week by specialization.

Overall, there was a significant difference between the three professional groups analyzed regarding the frequency of treating patients with a trauma diagnosis (Chi^2^ = 19.5, *p* = 0.003). Pediatrics emerged as the specialty with the highest number of treatments in the ‘1–5/week’ category, followed by trauma surgery (Figure 2).

### 3.2. Utilisation of Telemedical Cooperation

**Figure 3:**
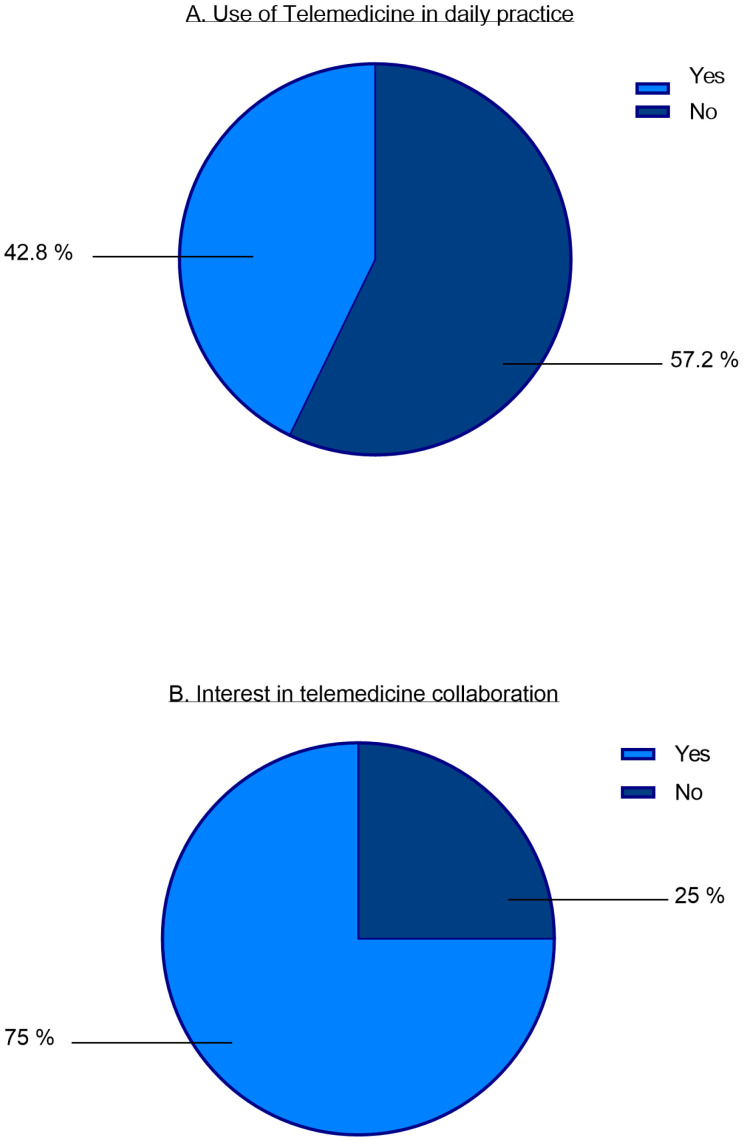
Telemedicine in daily practice.

Regarding the willingness and previous use of telemedicine interventions, the survey revealed that telemedicine is not yet widely used in daily practice with only 42.8% reporting usage (Figure 3A).

In general, 75% of all participants expressed interest in telemedicine collaboration with our clinic (Figure 3B). No significant gender-specific differences were found in this regard (Chi^2^ = 0.847 *p* = 0.357). Additionally, there were no significant differences in the general willingness to cooperate depending on the age groups of the participants surveyed (Chi^2^ = 2.936, *p* = 402). However, the results indicate that participants aged between 40 and 50 were significantly more willing to use messenger services than other age groups in terms of their preferred method of communication. Furthermore, there were no significant differences in specialization of the participants regarding the interest in telemedicine collaboration (Chi^2^ = 4.528, *p* = 0.104).

Physicians who already had previous experience with telemedicine showed a significantly higher interest in collaboration (Chi^2^ = 6.755, *p* = 0.009). The study revealed an overall difference in interest in telemedical cooperation across the investigated federal states, with participants from Hamburg showing the highest level of interest (Chi^2^ = 8.436, *p* = 0.038). However, no significant differences were observed in the distance from the practice to the nearest hospital.

No significant differences were found between private and group practices or outpatient health centers (Chi^2^ = 4.764, *p* = 0.190). Also, no significant differences were found between age groups concerning the possible use of imaging programs (Chi^2^ = 4.649, *p* = 0.199) or video telephony (Chi^2^ = 0.513, *p* = 0.916). Similarly, no differences were found between different specializations with regard to the possible use of a messaging service (Chi^2^ = 1.148, *p* = 0.563). In contrast, pediatricians used video telephony significantly more often than other specialties (Chi^2^ = 7.734, *p* = 0.021), whereas trauma surgeons were significantly more likely to use image processing software (Chi^2^ = 30.730, *p* < 0.001).

Preferences for a possible telemedicine approach varied. It was found that with the option of multiple choice, almost half of the respondents favoured telemedical collaboration via video telephony (49.4%). In the survey, 31.7% of respondents chose imaging applications like PACS (Picture Archiving Communication System), while 22.7% of votes chose messaging applications.

**Figure 4:**
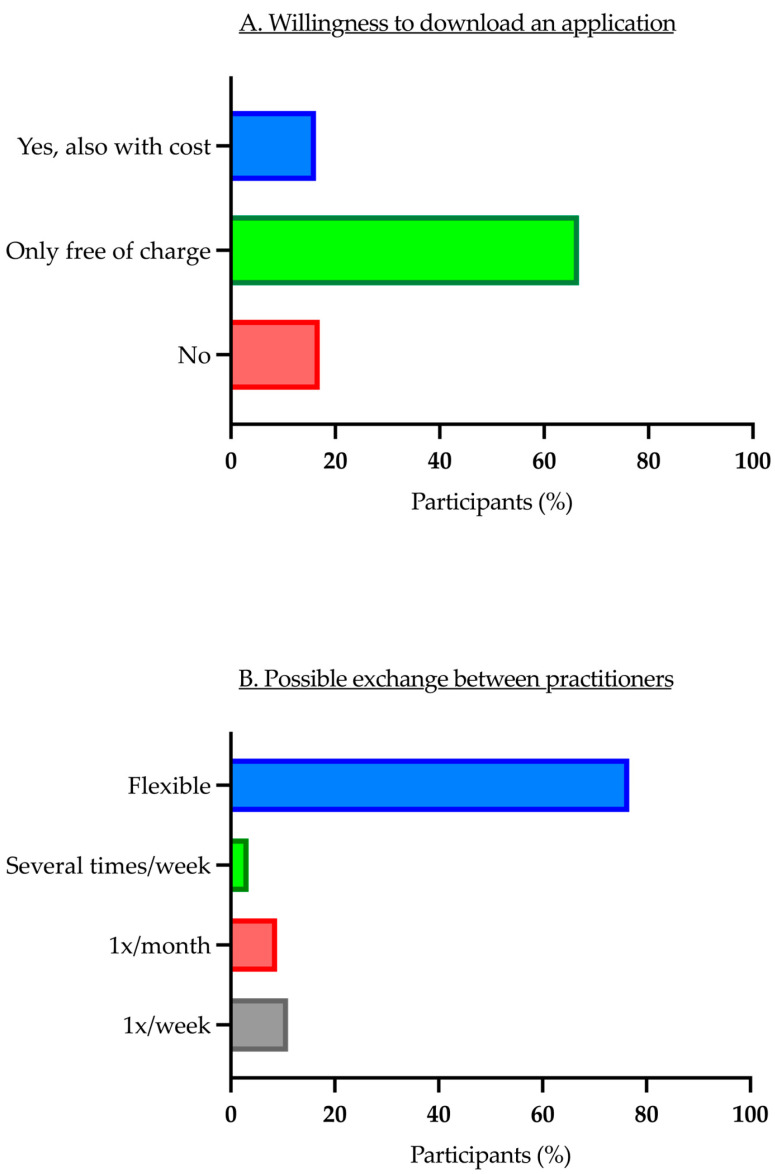
Application-download and exchange between practitioners.

The majority of respondents (83%) were willing to download applications for their telecommunication device. However, only 16.3% were willing to purchase applications, while 66.7% of the respondents were not willing to pay for them (Figure 4A).

Regarding potential communication between resident practicing physicians and the pediatric traumatology department, 76.7% of those surveyed were in favor of flexible communication. Of the participants, 11% preferred to communicate once a week, 8.9% once a month and only 3.4% several times a week (Figure 4B).

### 3.3. Reasons for Referral

**Figure 5:**
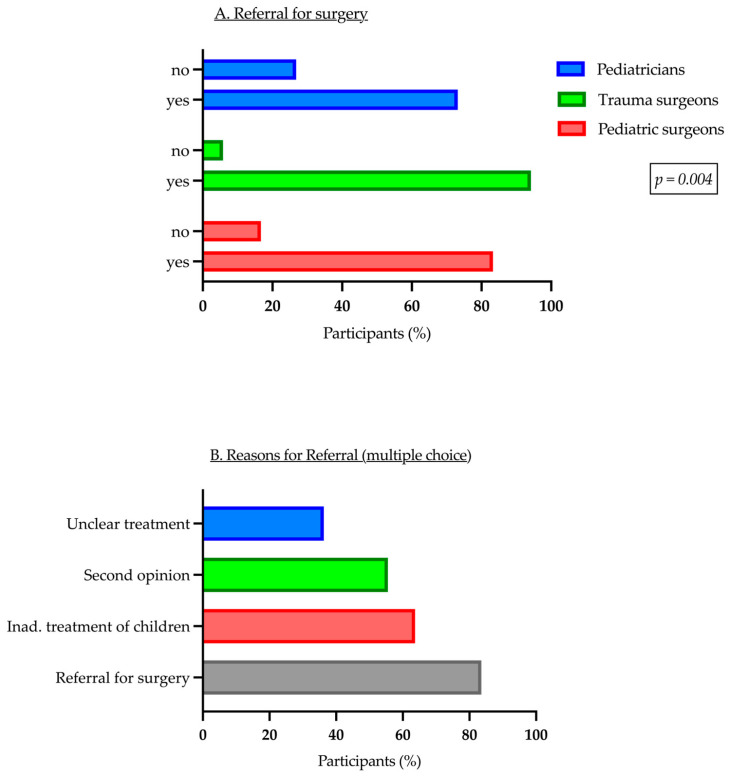
Referral to the hospital.

The main reason for referring patients to a hospital with expertise in pediatric trauma was the need for surgery, as reported by 83.4% of respondents. There was a notable disparity in patient referrals for surgery among the different specialties in our collective (*p* = 0.04) (Figure 5A). This was followed by inadequate treatment facilities for children in their practice, as reported by 60.4% of respondents. Furthermore, 52.6% of the group referred the patient for collaborative assessment, and in 34.4% of cases, there was uncertainty regarding the appropriate therapy (Figure 5B). The survey observed a significant disparity in patient referrals for surgery among the different specialties in the collective (Chi^2^ = 11.196, *p* = 0.04). Trauma surgeons (94.2%) and pediatric surgeons (83.3%) referred patients for surgery more frequently than pediatricians (73.2%). Upon further investigation into the reasons for referral, it becomes evident that there is a significant difference in patient referrals related to inadequate treatment (Chi^2^ = 37.740, *p* < 0.001) and those for co-assessment (Chi^2^ = 12.317, *p* = 0.002). In this context, our analyses demonstrate that trauma surgeons referred more patients for co-assessment (69.6%) than other specialties, while pediatricians more often referred their trauma patients due to inadequate treatment options (88.7%) than trauma or pediatric surgeons.

Referring patients due to unclear forms of therapy did not significantly differ across specialties (Chi^2^ = 1.897, *p* = 0.387). Furthermore, the professional experience of the participants did not affect the various reasons for referring patients to the hospital (all analyses: *p* > 0.05).

## 4. Discussion

The aim of our study was to investigate the interest of telemedical collaboration between private practices and the clinical sector in pediatric trauma surgery and to assess its potential benefits.

Despite the increasing trend towards telemedicine, our study found that only slightly more than half of our participants use telemedicine on a daily basis [13]. Despite the potential benefits for young patients and parents, pediatric medicine lags behind due to physician hesitation and other limitations like compensation or workflow disruption [11]. For this reason, there is a promising opportunity to enhance the use of telemedicine and accelerate the digitalization of the treatment process in pediatric healthcare.

There have already been significant scientific advances in the use of telemedicine in the field of traumatology. Sinha et al. compared telemedicine follow-up for pediatric fractures with regular hospital follow-up. The result indicated that the intervention group had demonstrated lower costs for parents/guardians, including reduced travel expenses and fewer days missed from work or school. Since pediatric patients typically do not attend medical appointments alone, hospital visits can result in dual absences from daycare or school and work, potentially leading to lost earnings and additional travel expenses [11].

In addition, practitioners were able to consult an average of 12 to 16 more patients than during regular follow-up [9]. Additionally, Dittrich et al. described that apps in trauma surgery are already widely utilized in hospitals and are frequently recommended to others [20]. Another recent study demonstrated a high level of acceptance and interest in the use of medical apps within the field of traumatology in the clinical field [16].

Accordingly, our survey revealed a strong willingness to engage in telemedicine collaboration, aligning with current medical developments and recent studies. Telemedicine has evolved into a practical means of maintaining and supporting medical care, offering potential cost reduction for hospitals and decreasing time demands for practitioners [13,20,21,22,23]. Moreover, telemedicine has been shown to reduce the workload in emergency departments [24].

However, to the best of our knowledge, there is no study yet that investigated the integration of telemedicine between the outpatient and inpatient sectors in the field of pediatric traumatology.

Our study results indicate that a slight majority of our respondents were pediatricians. While we did not observe a significant difference regarding potential telemedical collaboration, we were able to show that pediatricians significantly favoured communication via video telephony. Since traumatology is outside the scope of the actual specialization of pediatricians, the need for consultation can be inferred from this preference. In contrast, trauma surgeons significantly favoured exchange via image processing software. This is probably due to existing expertise, which only requires a focused exchange of information.

We were able to show that 66.7% of respondents were unwilling to pay for a necessary application. This reluctance may ultimately depend on the final cost of the program and its integration into the workflow. Nonetheless, our findings align with some of the acknowledged concerns of professionals [11,16].

Given that conservative and surgical treatments for trauma require acute decision-making, it is not surprising that most of the respondents advocate for flexible communication with the clinical sector. No significant age-related differences in telemedicine cooperation were observed. However, a recent study indicated that residents and senior physicians showed a particular interest [16]. Our results may be attributed to the fact that only 2.6% of respondents were between 30 and 40 years old, with the majority being older. Residents in hospitals are often under 30 years old and may as part of the ‘digital native’ generation be more inclined towards technology.

Additionally, a telehealth partnership can address two of the main reasons for hospital admissions identified in our survey. Initial evaluations for potential surgery and desired co-assessments of trauma patients, especially in cases of radiologically confirmed fractures, could be conducted via telemedicine. Subsequently, this allows for patient treatment based on the findings. In addition, if the treatment plan is unclear, advice can also be provided via an established communication channel, thereby reducing the burden on clinicians [9].

Interestingly, our study found no significant difference in the willingness to collaborate when comparing the distance between the practice and the nearest hospital with a pediatric trauma unit. Notably, participants from Hamburg were significantly more willing to participate in telemedicine collaboration. In contrast, many of the previous studies with pediatric patients have focused on the use of telemedicine in rural areas [23,24,25]. However, it is possible that the higher density of physicians in Hamburg may have influenced the result, as a large proportion of the respondents worked there. Nevertheless, these findings suggest that professional exchange with pediatric trauma specialists is vital, regardless of whether the practice is located in urban or rural areas.

Understandably, interest in telemedical cooperation with the clinic was significantly higher among participants already using telemedicine in their practice. Since the reasons for refusal were not explored in this study, further investigation and intervention are necessary to provide better information and improve willingness.

In our opinion, video telephony may not be suitable for spontaneous exchanges in daily work routines. However, specialized applications developed for medical professionals, such as the “Doctolib Siilo” app (Version 8.13.0, Siilo Holding B.V., Amsterdam, Netherlands), can serve as a communication tool. This has already been investigated in recent studies [26,27]. If required, healthcare professionals can still resort to personal phone or video calls.

## 5. Limitations

Overall, 154 out of the total contacted physicians responded to our survey; this corresponds to a return rate of 19.3%. This response rate is generally considered to be relatively low, possibly due to the anonymous nature of the survey or the lack of a reminder letter. It has also been shown that surveys of medical professionals generally have a lower response rate than those of non-medical professionals [28]. It should be noted that the study design is potentially susceptible to selection bias, given the participation of the responding doctors [29]. Furthermore, our study might not be representative, as we only contacted those practitioners who were registered on our database. Furthermore, we limited contact to practices within a 100 km radius from Hamburg. Therefore, our study does not represent a nationwide survey. Moreover, our questionnaire contained only 15 items; it serves as a cursory opinion survey, so more detailed follow-up studies are required.

## 6. Conclusions

There has already been evidence that the use of telemedicine can be a supportive tool in medicine to the benefit of both physicians and patients [10,13]. Within the pediatric traumatology outpatient sector, our findings revealed a substantial interest in telemedicine collaboration. Collaboration between outpatient clinics and hospitals in telemedicine can significantly reduce treatment redundancy, thereby lowering duplicate costs and unnecessary administrative burdens on health authorities. This is especially crucial given the growing scarcity of doctors and limited hospital resources [17,18]. The exchange of information should be tailored to meet individual needs, providing practitioners with a versatile and personalized approach that includes the capability for image processing. It is imperative that data protection regulations are strictly adhered to.

Further prospective research is required to evaluate the effectiveness of telemedicine collaboration in treating pediatric trauma in both hospital and outpatient settings. Ultimately, foreign healthcare systems that have similar treatment processes and comparable problems at the interface between the outpatient and inpatient sectors can adopt implemented telemedical cooperation.

## Figures and Tables

**Table 1:** Demographic and work-specific characteristics of the respondents.

	n (%)
Gender	151 (100)
Male	95 (62.9)
Female	56 (37.1)
Age (years)	154 (100)
30–40	4 (2.6)
40–50	80 (52.0)
50–60	28 (18.2)
>60	42 (27.3)
Profession	154 (100)
Trauma surgeons	71 (46.1)
Pediatrician	77 (50.0)
Pediatric surgeons	6 (3.9)
Professional experience (years)	154 (100)
5–10	5 (3.3)
10–20	51 (33.1)
>20	98 (63.6)
Workplace	154 (100)
Private practice	66 (42.9)
Group practice	64 (41.6)
Outpatient health center	23 (14.9)
Other	1 (0.7)
Location/Federal State	152 (100)
Hamburg	99 (65.1)
Lower Saxony	19 (12.5)
Schleswig-Holstein	32 (21.1)
Mecklenburg–Western Pomerania	2 (1.3)
Distance to the nearest traumatologic department (km)	153 (100)
<5	7 (4.6)
5–10	99 (64.7)
10–25	33 (21.6)
25–50	10 (6.5)
>50	4 (2.6)
No. of ped. traumatologic patients treated/week	152 (100)
1–5	92 (60.5)
5–15	27 (17.8)
>15	15 (9.9)
None	18 (11.8)

Missing data were excluded from descriptive statistics.

## Data Availability

The raw data supporting the conclusions of this article will be made available by the authors upon request.

## Supplementary Materials

The following supporting information can be downloaded at: https://www.mdpi.com/article/10.3390/children11091120/s1, S1: Telemedicine outpatient survey.
