# Supplementary material for: Enhancing Telemedicine Communication for Improved Outpatient Pediatric Trauma Care
Source: Children (Basel). 2024 Sep 12;11(9):1120. doi: 10.3390/children11091120 (PMC11429903; doi:10.3390/children11091120)
Supplement: Supplementary file 1 [file children-11-01120-s001.zip › children-3181646-supplementary.docx]

Telemedicine Collaboration in Paediatric Trauma Patients

1. Sex:

a. Female

b. Male

c. Diverse

2. Specialty/Additional qualification (multiple answers possible)

a. Pediatrics

b. Trauma surgery and orthopaedics

c. Paediatric surgery

d. Paediatric orthopaedics

e. Specialised trauma surgery

3. Age of participant?

a. 20-30 years

b. 30-40 years

c. 40-50 years

d. 60 years

4. In which federal state is your practice located?

a. Hamburg

b. Lower Saxony

c. Schleswig-Holstein

d. Mecklenburg–Western Pomerania

5. Distance to the nearest hospital with (paediatric) traumatology treatment facilities?

a. 5-10 km

b. 10-25 km

c. 25-50 km

d. >50 km

6. Professional experience of the participant?

a. < 5 years

b. 5-10 years

c. 10-20 years

d. >20 years

7. Form of practice:

a. Private practice

b. MVZ

c. Group practice

8. Treatment of pediatric traumatology patients per week?

a. None

b. 1-5 patients/week

c. 5-15 patients/week

d. > 15 patients/week

9. Use of mobile devices in private everyday life/practice (multiple choice)?

a. Mobile phone

b. Tablet

c. Laptop

d. None

10. Prior telemedicine experience/use in everyday practice?

a. Yes

b. No

11. Preferred telemedical medium for pediatric traumatology issues?

a. Messenger service (Silo, etc.)

b. Video chat/telephone call

c. Image processing program (e.g., PACS)

12. Willingness to download software/applications?

a. Yes, but only free of charge

b. Yes, also for a fee

c. No

13. Favoured frequency of exchange?

a. Once a week

b. Several times a week

c. Once a month

d. Flexible

14. Reasons for referral to hospital/specialist?

a. Need for surgery

b. Second opinion

c. Unclear form of therapy

d. Insufficient treatment options in the practice (X-ray diagnostics, materials) in the care of children

15. General interest in telemedicine collaboration?

a. Yes

b. No
